# Geriatric Nutritional Risk Index as a Tool to Evaluate Impact of Malnutrition Risk on Mortality in Adult Patients with Polytrauma

**DOI:** 10.3390/ijerph17249233

**Published:** 2020-12-10

**Authors:** Cheng-Hsi Yeh, Shao-Chun Wu, Sheng-En Chou, Wei-Ti Su, Ching-Hua Tsai, Chi Li, Shiun-Yuan Hsu, Ching-Hua Hsieh

**Affiliations:** 1 Department of General Surgery, Kaohsiung Chang Gung Memorial Hospital, College of Medicine, Chang Gung University, Kaohsiung City 83301, Taiwan; ycc9002108@hotmail.com; 2 Department of Anesthesiology, Kaohsiung Chang Gung Memorial Hospital, College of Medicine, Chang Gung University, Kaohsiung City 83301, Taiwan; shaochunwu@gmail.com; 3 Department of Trauma Surgery, Kaohsiung Chang Gung Memorial Hospital, College of Medicine, Chang Gung University, Kaohsiung City 83301, Taiwan; athenechou@gmail.com (S.-E.C.); s101132@adm.cgmh.org.tw (W.-T.S.); tsai1737@cloud.cgmh.org.tw (C.-H.T.); foocollie7@gmail.com (C.L.); ah.lucy@hotmail.com (S.-Y.H.); 4 Department of Plastic Surgery, Kaohsiung Chang Gung Memorial Hospital, College of Medicine, Chang Gung University, Kaohsiung City 83301, Taiwan

**Keywords:** malnutrition, mortality, geriatric nutritional risk index (GNRI), trauma, polytrauma

## Abstract

Background: Identification of malnutrition is especially important in severely injured patients, in whom hypermetabolism and protein catabolism following traumatic injury worsen their nutritional condition. The geriatric nutritional risk index (GNRI), based on serum albumin level and the current body weight/ideal body weight ratio, is useful for identifying patients with malnutrition in many clinical conditions. This study aimed to explore the association between admission GNRI and mortality outcomes of adult patients with polytrauma. Methods: From 1 January 2009 to 31 December 2019, a total of 348 adult patients with polytrauma, registered in the trauma database of a level I trauma center, were recognized and categorized into groups of death (*n* = 71) or survival (*n* = 277) and into four nutritional risk groups: a high-risk group (GNRI < 82, *n* = 87), a moderate-risk group (GNRI 82 to <92, *n* = 144), a low-risk group (GNRI 92–98, *n* = 59), and a no-risk group (GNRI > 98, *n* = 58). Univariate and multivariate logistic regression analyses were used to identify the independent risk factors for mortality. The mortality outcomes of patients at various nutritional risks were compared to those of patients in the no-risk group. Results: The comparison between the death group (*n* = 71) and the survival group (*n* = 277) revealed that there was no significant difference in gender predominance, age, pre-existing comorbidities, injury mechanism, systolic blood pressure, and respiratory rate upon arrival at the emergency room. A significantly lower GNRI and Glasgow Coma Scale score but higher injury severity score (ISS) was observed in the death group than in the survival group. Multivariate logistic regression analysis revealed that Glasgow Coma Scale (GCS), odds ratio (OR), 0.88; 95% confidence interval (CI), 0.83–0.95; *p* < 0.001), ISS (OR, 1.07; 95% CI, 1.04–1.11; *p* < 0.001), and GNRI (OR, 0.94; 95% CI, 0.91–0.97; *p* < 0.001) were significant independent risk factors for mortality in these patients. The mortality rates for the high-risk, moderate-risk, low-risk, and no-risk groups were 34.5%, 20.1%, 8.5%, and 12.1%, respectively. Unlike patients in the moderate-risk and low-risk groups, patients in the high-risk group had a significantly higher death rate than that of those in the no-risk group. Conclusions: This study revealed that the GNRI may serve as a simple, promising screening tool to identify the high risk of malnutrition for mortality in adult patients with polytrauma.

## 1. Background

A prevalence of malnutrition ranging from 7% to 76% has been reported in severely injured patients [1]. Furthermore, following traumatic injury, metabolic responses such as hypermetabolism and marked protein catabolism worsen the nutritional condition of trauma patients [2,3]. Unabated malnutrition during critical illness leads to impaired immunity, increased infections, and worsened survival [4,5]. Therefore, it is important to implement nutrition therapy and employ it continuously for these critically ill trauma patients. However, although currently, a great diversity of nutritional screening and assessment tools are available, there is no gold standard for nutritional assessment [6], especially in severely injured trauma patients.

Current clinical practice guidelines prefer the use of serum albumin levels to assess and monitor nutritional status [7,8,9,10]. However, the serum albumin level is affected by the hydration status of the patient, the inflammatory process, and the impairment of hepatic or renal functions. Therefore, it does not accurately reflect the nutritional status of critically ill patients [11]. In 2005, Bouillanne et al. introduced the geriatric nutritional risk index (GNRI), which is calculated as 1.489 × albumin (g/L) + 41.7 × (current body weight/ideal body weight) to assess nutritional status [12]. Current body weight/ideal body weight ratios which are equal or greater than one, are written as 1 [12]. According to the GNRI score, the patients were divided into four groups with different nutritional risks: a no-risk group (GNRI > 98), a low-risk group (GNRI 92–98), a moderate-risk group (GNRI 82 to <92), and a high-risk group (GNRI < 82). The risk of infectious complications or mortality was significantly higher in the major-, moderate-, and low-risk groups than in the no-risk group [12]. With additional information on current body weight/ideal body weight, the GNRI demonstrated a higher predictive performance of mortality than that of albumin level alone [13] and correlated well with the circumference of the mid-upper arm muscle [14], other nutritional scoring methods [15,16], and the preoperative sarcopenia status of patients [17]. Although the GNRI was first developed to evaluate the 6-month midterm nutritional outcomes of elderly patients in the rehabilitation unit [12], it proved to be a simple tool to assess long-term postoperative outcomes [18,19,20,21,22] as well as many other medical conditions including sepsis [23], heart failure [24], chronic obstructive pulmonary disease [25], chronic renal disease [26], and malignancies [27,28].

Some studies have reported the use of the GNRI has not been limited to geriatric patients [29,30,31,32,33]. However, no study on GNRI has yet been undertaken in the trauma population, specifically in those who have polytrauma. Seeing that polytrauma is a critical condition in which nutritional status may play an important role in a patient’s survival, therefore, this study was designed to identify the association between admission GNRI and outcomes of adult trauma patients with polytrauma. This study was performed according to the analysis of retrospectively collected data from the registered trauma database of a level I trauma center.

## 2. Methods

### 2.1. Study Population and Data Collection

**Figure 1:**
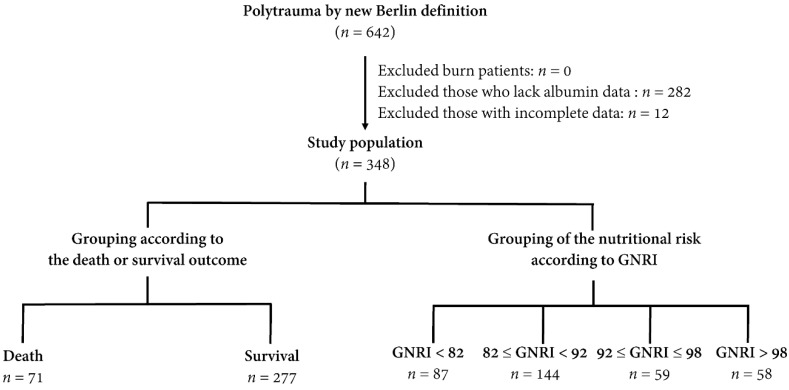
Flowchart illustrating the inclusion of adult patients with polytrauma from the Trauma Registry System, with the allocation of these patients into groups of death and survival as well as groups of four nutritional risk groups according to the geriatric nutritional risk index (GNRI).

The study was approved by the Institutional Review Board (IRB) of the Chang Gung Memorial Hospital (approval number 202001446B0) before implementation. According to IRB regulations, the requirement for informed consent was waived due to the retrospective nature of this study. Since the commonly used definition of polytrauma, which is abbreviated injury scale (AIS) ≥ 3 for at least two body regions, failed to recognize a significant difference in short-term mortality among trauma patients [34], in this study, polytrauma was defined on the basis of the new Berlin definition as follows [35]: AIS ≥ 3 for two or more different body regions with one or more additional variables from the five described physiologic parameters. These parameters included systolic blood pressure (SBP) ≤ 90 mm Hg, Glasgow Coma Scale (GCS) score ≤ 8, base excess ≤ 6.0, international normalized ratio ≥ 1.4 or partial thromboplastin time ≥ 40 s, and ≥70 years of age. In the Trauma Registry System, 642 adult patients (aged ≥ 20 years) had sustained polytrauma. After excluding patients with burns (*n* = 0), patients lacking albumin data (*n* = 282), and patients with incomplete data (*n* = 12), 348 adult patients with polytrauma were enrolled in the study population (Figure 1). The study population was categorized into groups of patients according to the final outcome as death (*n* = 71) or survival (*n* = 277) and according to the nutritional risks by the GNRI. The latter was divided into four nutritional risk groups (group 1, a high-risk group, GNRI < 82; group 2, a moderate-risk group, GNRI 82 to <92; group 3, a low-risk group, GNRI 92–98; and group 4, a no-risk group, GNRI > 98) according to the recommendations taken from Bouillanne et al. [12]. The patients’ medical information was collected from the Trauma Registry System of the hospital [34,36,37]. This included sex, age, preexisting comorbidities (diabetes mellitus (DM), hypertension (HTN), coronary artery disease (CAD), and end-stage renal disease (ESRD)), injury mechanism (blunt or penetrating injury), SBP, respiratory rate (RR) upon arrival at the emergency room, GCS score, injury severity score (ISS), serum albumin levels (g/dL) on admission, body mass index (BMI), length of stay (LOS) in hospital (days), and in-hospital mortality.

### 2.2. Statistical Analyses

The normalization of the distributed data for continuous variables was analyzed using the Kolmogorov–Smirnov test. Analysis of variance was used with the Bonferroni post hoc correction to analyze continuous data with a normal distribution. The non-normally distributed continuous data were analyzed using the Mann–Whitney *U*-test. The results are expressed as mean ± standard deviation or median with interquartile range (IQR, Q1–Q3). Categorical data were compared using the two-sided Fisher’s exact test or Pearson’s χ^2^ test. Univariate predictive variables that resulted in patient mortality were identified, and multivariate logistic regression analysis was used to identify independent risk factors for mortality, with the presentation of odds ratios (ORs) and 95% confidence intervals (CIs). The in-hospital mortality of patients was defined as the primary outcome. All statistical analyses were performed using Windows version 23.0 for SPSS (IBM Inc., Chicago, IL, USA). *p* values <0.05 indicated statistical significance.

## 3. Results

### 3.1. Patient and Injury Characteristics of the Death and Survival Groups of Patients

As shown in Table 1, the comparison between the death group (*n* = 71) and survival group (*n* = 277) of the study population revealed that there was no significant difference in gender predominance, age, pre-existing co-morbidities, injury mechanism, SBP, RR, and BMI upon arrival at the emergency room. A significantly lower GCS was observed in the death group than in the survival group (median IQR: 5 (3–8) vs. 11 (6–15), respectively; *p* < 0.001). When stratified by GCS (3–8, 9–12, or 13–15), significantly fewer patients had scores of 13–15, but more had scores of 3–8 in the death group than in the survival group. A significantly higher ISS was observed in the death group than in the survival group (median IQR: 35 (29–41) vs. 29 (22–34), respectively; *p* < 0.001). When stratified by ISS (16–24 or ≥25), there were significantly more patients with an ISS of ≥25, but fewer dead patients than patients that survived, with scores of 16–24. The death group presented a significantly lower level of albumin (2.7 ± 0.8 vs. 3.2 ± 0.7 g/dL; *p* < 0.001) and GNRI (83.0 ± 10.4 vs. 89.0 ± 9.1, respectively; *p* < 0.001) than the survival group did. Patients in the death group had a significantly shorter hospital LOS (14.7 days vs. 29.5 days, respectively; *p* < 0.001) than those in the survival group did.

### 3.2. Analysis of the Risk Factors for Mortality

Univariate analysis revealed that the GCS, ISS, and GNRI were significant risk factors for mortality in adult patients with polytrauma (Table 2). Subsequent multivariate logistic regression analysis revealed that GCS (OR, 0.88; 95% CI, 0.83–0.95; *p* < 0.001), ISS (OR, 1.07; 95% CI, 1.04–1.11; *p* < 0.001) and GNRI (OR, 0.94; 95% CI, 0.91–0.97; *p* < 0.001) were significant independent risk factors for mortality in these patients. In addition, gender, age, comorbidities (DM, HTN CAD) were not risk factors for mortality.

### 3.3. Patient and Injury Characteristics of the Patients with Different Nutritional Risks

According to the GNRI score, there were 87, 144, 59, and 58 patients allocated in group 1 (high-risk), group 2 (moderate-risk), group 3 (low-risk), and group 4 (no-risk), respectively (Table 3). There was a significantly higher percentage of male patients in group 1 than in group 4. There was no significant difference in age, preexisting comorbidities, GCS, and ISS among the groups with different risks for malnutrition. Patients in group 1 (34.5%), but not in groups 2 (20.1%) and 3 (8.5%), had a significantly higher death rate than patients in group 4 (12.1%). There was no significant difference in hospital LOS among these groups with different risks for malnutrition.

## 4. Discussion

This study revealed that in adult patients with polytrauma, the death group presented a significantly lower GNRI (83.0 ± 10.4 vs. 89.0 ± 9.1, respectively; *p* < 0.001) than the survival group did. In addition, multivariate logistic regression analysis identified the GNRI as a significant independent risk factor for mortality. Unlike moderate-risk and low-risk patients, a significantly higher mortality rate was observed in high-risk patients than in non-risk patients. This was due to the minor odds of risk for mortality influenced by the GNRI (OR 0.94; 95% CI, 0.91–0.97). Currently, the most commonly used prediction algorithm for mortality outcomes in trauma patients is the Trauma and Injury Severity Score (TRISS) [38,39,40]. The TRISS determines the probability of survival by four variables: age, ISS, Revised Trauma Score (RTS), and injury mechanisms such as blunt or penetrating injuries. ISS is an anatomical variable that indicates the severity of injury, while RTS is a physiological variable value regarding the patient’s initial GCS score, SBP, and RR [41]. Although nutrition is important for the care of severe trauma patients [42], and early enteral nutrition reduces the length of hospital stay and mortality [43], nutritional status is not considered while calculating TRISS. The GNRI has been reported to correlate well with the overall complications of the patients, especially that of death [44,45]. Since death is a terminal condition that might be a consequence of nutrition-related complications in trauma patients [46,47], it may be valuable to incorporate nutritional status into the mortality prediction algorithm.

The GNRI was associated with mortality in several clinical conditions. In patients with peripheral artery diseases, the GNRI served as a predictor of overall survival and major adverse cardiovascular events with or without limb events [48]. In patients undergoing peritoneal dialysis, the GNRI is an independent predictor of mortality [31]. In patients undergoing chronic hemodialysis, a low GNRI was associated with cardiovascular mortality (adjusted hazard ratios, 1.93; 95% CI, 1.1–4.8) and all-cause mortality (adjusted hazard ratios, 1.85; 95% CI, 1.1–3.2) [33]. In patients with pyogenic liver abscess, the GNRI presents a better predictive performance than BMI, albumin level, platelet count, prothrombin time, and hemoglobin level in mortality and all adverse outcomes [49]. This study revealed that the GNRI may serve as a promising simple screening tool to identify subjects with high-risk malnutrition in patients with polytrauma. The GNRI can be easily acquired on the basis of the sex, height, weight, and serum albumin levels of patients. For polytrauma patients who have difficulties in communicating, such as those with conscious disturbance or those under intubation, the advantages of using the GNRI is that it would supersede traditional questionnaires, including the subjective global assessment (SGA) [50] or Mini Nutritional Assessment (MNA) [51].

Whether the GNRI could also be used for nutritional management for these patients is interesting but requires further investigation to validate. Notably, this study has some limitations. First, there may have been a selection bias due to the retrospective nature of the study. Additionally, data of patients that were declared dead upon arrival at the emergency room were not recorded in the registered database. Furthermore, long-term mortality was not evaluated in this study since data were collected on in-hospital stays only. Both conditions may have led to a selection bias in the mortality outcome measurement. Moreover, another selection bias may exist owing to the exclusion of many patients without albumin level data. Another limitation arises from the fact that interventions such as resuscitation, damage control, and surgery could lead to a different outcome for the patients in question. However, in this study, we can only assume that the outcome of these interventions was uniform across the studied population. Furthermore, it should be noted that the disadvantages of the non-parametric test are that it is less efficient as compared to the parametric test and the results may or may not provide an accurate answer due to distribution-free aspect of the data. Finally, the population included in this study was limited to that from a single urban trauma center; thus, these results may not be generalizable to other regions.

## 5. Conclusions

This study revealed that the GNRI may serve as a simple, promising screening tool to identify high-risk malnutrition for mortality in adult patients with polytrauma.

## Figures and Tables

**Table 1:** Patient and injury characteristics of the death and survival groups of adult patients with polytrauma.

Variables	Death *n* = 71		Survival *n* = 277		*p*
Gender					0.519
Male, *n* (%)	52	(73.2)	192	(69.3)	
Female, *n* (%)	19	(26.8)	85	(30.7)	
Age (years)	56.4	±21.9	54.7	±19.5	0.522
Co-morbidities					
DM, *n* (%)	14	(19.7)	43	(15.5)	0.394
HTN, *n* (%)	16	(22.5)	81	(29.2)	0.261
CAD, *n* (%)	6	(8.5)	13	(4.7)	0.214
ESRD, *n* (%)	0	(0.0)	3	(0.1)	-
Injury mechanism					0.378
Blunt, *n* (%)	71	(100.0)	274	(98.9)	
Penetrating, *n* (%)	0	(0.0)	3	(1.1)	
SBP (mmHg)	122.1	±55.1	119.9	±41.3	0.706
RR (times/min)	19.9	±7.1	20.4	±5.6	0.516
GCS, median (IQR)	5	(3–8)	11	(6–15)	<0.001
3–8, *n* (%)	54	(76.1)	123	(44.4)	<0.001
9–12, *n* (%)	4	(5.6)	34	(12.3)	0.109
13–15, *n* (%)	13	(18.3)	120	(43.3)	<0.001
ISS, median (IQR)	35	(29–41)	29	(22–34)	<0.001
16–24, *n* (%)	4	(5.6)	81	(29.2)	<0.001
≥25, *n* (%)	67	(94.4)	196	(70.8)	<0.001
Albumin (g/dL)	2.7	±0.8	3.2	±0.7	<0.001
BMI	25.4	±4.7	25.2	±4.4	0.673
GNRI	83.0	±10.4	89.0	±9.1	<0.001
LOS in hospital (days)	14.7	±18.7	29.5	±17.6	<0.001

BMI = body mass index; CAD = coronary artery disease; CI = confidence interval; DM = diabetes mellitus; ESRD = end-stage renal disease; GCS = Glasgow Coma Scale; GNRI = geriatric nutritional risk index; HTN = hypertension; IQR = interquartile range; ISS = injury severity score; LOS = length of stay; OR = odds ratio; RR = respiratory rate; SBP = systolic blood pressure.

**Table 2:** Univariate and multivariate analysis of the risk factors for mortality.

Variables	Univariate Analysis			Multivariate Analysis		
	OR (95% CI)		*p*	OR (95% CI)		*p*
Gender	1.2	(0.68–2.17)	0.520	1.2	(0.62–2.50)	0.538
Age	1.0	(0.99–1.02)	0.521	1.0	(0.99–1.04)	0.068
DM	1.3	(0.69–2.61)	0.395	2.2	(0.93–5.18)	0.072
HTN	0.7	(0.38–1.30)	0.262	0.5	(0.24–1.18)	0.119
CAD	1.9	(0.69–5.12)	0.220	1.7	(0.48–5.96)	0.408
GCS	0.85	(0.79–0.90)	<0.001	0.88	(0.83–0.95)	<0.001
ISS	1.09	(1.06–1.13)	<0.001	1.07	(1.04–1.11)	<0.001
GNRI	0.94	(0.91–0.96)	<0.001	0.94	(0.91–0.97)	<0.001

CI = confidence interval; GCS = Glasgow Coma Scale; GNRI = geriatric nutritional risk index; ISS = injury severity score; OR = odds ratio.

**Table 3:** Patient and injury characteristics of the adult polytrauma patients with different risks for malnutrition.

Variables	Group 1	Group 2	Group 3	Group 4	*p*
	*n* = 87	*n* = 144	*n* = 59	*n* = 58	
Gender					0.009
Male, *n* (%)	51 (58.6) *	99 (68.9)	47 (79.7)	47 (81.0)	
Female, *n* (%)	36 (41.4) *	45 (31.2)	12 (20.3)	11 (19.0)	
Age (years)	56.2 ± 19.7	57.0 ± 19.9	51.3 ± 19.6	52.3 ± 20.7	0.191
BMI	20.0 ± 4.8 *	25.3 ± 4.5	25.2 ± 4.0	26.9 ± 3.7	0.002
Co-morbidities					
DM, *n* (%)	10 (11.5)	29 (20.1)	10 (16.9)	8 (13.8)	0.348
HTN, *n* (%)	20 (23.0)	44 (30.6)	17 (28.8)	16 (27.6)	0.665
CAD, *n* (%)	4 (4.6)	8 (5.6)	6 (10.2)	1 (1.7)	0.238
ESRD, *n* (%)	0 (0.0)	3 (2.1)	0 (0.0)	0 (0.0)	0.232
GCS, median (IQR)	8 (3–15)	11 (6–15)	8 (5–15)	8 (5–15)	0.214
3–8, *n* (%)	48 (55.2)	65 (45.1)	32 (54.2)	32 (55.2)	0.357
9–12, *n* (%)	9 (10.3)	19 (13.2)	4 (6.8)	6 (10.3)	0.603
13–15, *n* (%)	30 (34.5)	60 (41.7)	23 (39.0)	20 (34.5)	0.660
ISS, median (IQR)	29 (25–38)	29 (25–36)	29 (25–36)	29 (22–34)	0.593
1–15, *n* (%)	0 (0.0)	0 (0.0)	0 (0.0)	0 (0.0)	-
16–24, *n* (%)	21 (24.1)	31 (21.5)	13 (22.0)	20 (34.5)	0.259
≥25, *n* (%)	66 (75.9)	113 (78.5)	46 (78.0)	38 (65.5)	0.259
Mortality, *n* (%)	30 (34.5) *	29 (20.1)	5 (8.5)	7 (12.1)	<0.001
LOS in hospital (days)	29.7 ± 24.4	26.1 ± 16.5	24.9 ± 13.6	24.3 ± 18.6	0.291

BMI = body mass index; CAD = coronary artery disease; DM = diabetes mellitus; ESRD = end-stage renal disease; GCS = Glasgow Coma Scale; HTN = hypertension; ISS = injury severity score; LOS = length of stay. * indicates a *p*-value <0.05 in comparison with group 4 patients.
